# Mother–Fetus Immune Cross-Talk Coordinates “Extrinsic”/“Intrinsic” Embryo Gene Expression Noise and Growth Stability

**DOI:** 10.3390/ijms232012467

**Published:** 2022-10-18

**Authors:** Tatyana Ivanovna Babochkina, Ludmila Alekseevna Gerlinskaya, Margarita Vladimirovna Anisimova, Galina Vladimirovna Kontsevaya, Natalia Aleksandrovna Feofanova, Aliya Konstantinovna Stanova, Mikhail Pavlovich Moshkin, Yuri Mikhailovich Moshkin

**Affiliations:** 1 Federal Research Center Institute of Cytology and Genetics, Siberian Branch of RAS, 630090 Novosibirsk, Russia; 2 Department of Zoology and Animal Ecology, Tomsk State University, 634050 Tomsk, Russia; 3 Gene Learning Association, 1205 Geneva, Switzerland

**Keywords:** developmental instability, fluctuating asymmetry, variance/noise, MHC

## Abstract

Developmental instability (DI) is thought to be inversely related to a capacity of an organism to buffer its development against random genetic and environmental perturbations. DI is represented by a trait’s inter- and intra-individual variabilities. The inter-individual variability (inversely referred to as canalization) indicates the capability of organisms to reproduce a trait from individual to individual. The intra-individual variability reflects an organism’s capability to stabilize a trait internally under the same conditions, and, for symmetric traits, it is expressed as fluctuating asymmetry (FA). When representing a trait as a random variable conditioned on environmental fluctuations, it is clear that, in statistical terms, the DI partitions into “extrinsic” (canalization) and “intrinsic” (FA) components of a trait’s variance/noise. We established a simple statistical framework to dissect both parts of a symmetric trait variance/noise using a PCA (principal component analysis) projection of the left/right measurements on eigenvectors followed by GAMLSS (generalized additive models for location scale and shape) modeling of eigenvalues. The first eigenvalue represents “extrinsic” and the second—“intrinsic” DI components. We applied this framework to investigate the impact of mother–fetus major histocompatibility complex (MHC)-mediated immune cross-talk on gene expression noise and developmental stability. We showed that “intrinsic” gene noise for the entire transcriptional landscape could be estimated from a small subset of randomly selected genes. Using a diagnostic set of genes, we found that allogeneic MHC combinations tended to decrease “extrinsic” and “intrinsic” gene noise in C57BL/6J embryos developing in the surrogate NOD-SCID and BALB/c mothers. The “intrinsic” gene noise was negatively correlated with growth (embryonic mass) and the levels of placental growth factor (PLGF), but not vascular endothelial growth factor (VEGF). However, it was positively associated with phenotypic growth instability and noise in PLGF. In mammals, the mother–fetus MHC interaction plays a significant role in development, contributing to the fitness of the offspring. Our results demonstrate that a positive impact of distant MHC combinations on embryonic growth could be mediated by the reduction of “intrinsic” gene noise followed by the developmental stabilization of growth.

## 1. Introduction

The random deviation of a phenotypic trait from bilateral symmetry is referred to as fluctuating asymmetry (FA). FA is thought to quantify the developmental instability (DI) caused by the insufficient buffering of random genetic and environmental perturbations within a developing organism. Similarly, but not interchangeably, DI could be expressed through between-individual variations in a phenotypic trait. The buffering of phenotypic fluctuation against environmental and genetic perturbations across individuals is also known as canalization [1,2,3,4]. FA and canalization could also be thought of as “intrinsic” and “extrinsic” variability, respectively. Such a distinction is now widely adopted to partition a fluctuation (variance, the coefficient of variation) in a given mRNA copy number/concentration, referred to as gene expression noise. The “intrinsic” perturbations in gene expression can be determined by evaluating stochastic changes in mRNA copy numbers between distinct alleles, while “extrinsic” ones correspond to between-cell or inter-individual variability [5,6,7,8]. From this reasoning, it is clear that the “intrinsic” component of gene expression noise can be estimated from FA without allelic information.

Strictly speaking, for open systems, such as biological ones, the terms “extrinsic” and “intrinsic” have no physical meaning and are used for mere convenience [9,10]. Indeed, both “extrinsic” fluctuation (canalization) and “intrinsic” (FA) reflect the ability of a biological system to buffer an impact of genetic perturbations and/or environmental flux, ξ, on a trait, x. In other words, a trait, x, is conditioned on the upstream (relative to x) drive ξ, and its variance ($\sigma_{x}^{2}$) is composed of “extrinsic” (variance of expectation—Var[E(x| ξ)]) and “intrinsic” (expectation of variance—E[Var(x| ξ)]) components:

$$\sigma_{x}^{2}=\underset{“\mathrm{extrinsic}”}{\underbrace{\mathrm{Var}[E(x|\;\xi )]}}+\underset{“\mathrm{intrinsic}”}{\underbrace{E[\mathrm{Var}\left(x|\;\xi \right)].}}$$

If a bilateral trait x is measured on the left (l) and right (r) sides, and assuming that $\sigma_{x}^{2}\approx \sigma_{l}^{2}\approx \sigma_{r}^{2}$, it can be readily shown that canalization corresponds to the “extrinsic” variance component:

(2) $$\underset{“\mathrm{extrinsic}”}{\underbrace{\mathrm{Var}[E(x|\;\xi )]}}=\mathrm{Var}\left(\frac{l+r}{2}\right)=\frac{1}{4}\sigma_{l+r}^{2}\approx \frac{1}{2}\sigma_{x}^{2}\left(1+\rho_{l,r}\right)$$

as $\sigma_{l+r}^{2}$ measures between-individual variability, i.e., canalization, $\rho_{l,r}$ indicates the Pearson correlation coefficient between l and r. From this,

(3) $$\underset{“\mathrm{intrinsic}”}{\underbrace{E\left[\mathrm{Var}\left(x|\;\xi \right)\right]}}\approx \frac{1}{4}\mathrm{FA}=\frac{1}{4}\sigma_{l-r}^{2}\approx \frac{1}{2}\sigma_{x}^{2}(1-\rho_{l,r}).$$

Defining FA as a variance of left/right differences ($\mathrm{FA}=\sigma_{l-r}^{2}$), it becomes clear that FA is a particular case of the “intrinsic” variance component. Although other measures of FA exist [11,12], this one has an unambiguous statistical interpretation. Thus, the canalization and FA could be viewed as the decomposition of DI ($\sigma_{x}^{2}$) into two intertwined components: “extrinsic” and “intrinsic”, respectively:

(4) $$\sigma_{x}^{2}\approx \frac{1}{4}\underset{“\mathrm{extrinsic}”}{\underbrace{\sigma_{l+r}^{2}}}+\frac{1}{4}\underset{“\mathrm{intrinsic}”}{\underbrace{\sigma_{l-r}^{2}}}.$$

Both bear information on fluctuations in the external drive ξ, smearing a clear-cut distinction between the terms “extrinsic” and “intrinsic” (Equation (1)).

As such, DI and its components (“extrinsic” and “intrinsic”) are population-wise estimates of a trait’s fluctuation. However, evaluating the FA for an ensemble of multiple traits/genes can measure the “intrinsic” developmental/transcriptional stability at an individual level [13]. As a result, ensemble FA allows for the investigation of mechanisms driving individual rather than populational stability. Composite FA is inversely related to individuals’ quality/fitness [14,15] and increases with age [16]. Estimating individuals’ developmental/transcriptional instability could also be practical for diagnostics, as some diseases, such as those that are infectious and inflammatory, destabilize the transcriptional landscape [17].

FA depends on genomic stability, epigenetic buffering, proteostasis, and growth control [18]. Indeed, in flies, the loss of Cyclin G increases FA and DI, likely due to a disruption of p53-dependent DNA repair [19]. Furthermore, epigenetic alterations caused by mutations in subunits of Polycomb-repressive complexes PRC1 and PR-DUB increase the FA in conjunction with *CycG* [20], indicating, on the one hand, that the Polycomb complexes are required to maintain genomic stability [21], while on the other hand, that PRC1 and PR-DUB are also involved in regulating transcriptional stability [22]. Thus, epigenetic homeostasis could buffer developmental fluctuations on both the genomic and transcriptional stability levels. Downstream of genomic and transcriptional stability, perturbations in protein quality homeostasis significantly impact DI. Molecular chaperones maintain proteostasis and, as a result, are required for developmental stability [23,24,25]. Finally, control over growth and cell fate determination is essential in establishing symmetry. In Drosophila, mutations in the insulin-like growth factor *Dilp8* and the *Notch* cell fate determination pathway increase FA [26,27].

FA tends to increase with decreasing genetic variability (heterozygosity) [2,4,28,29]. However, in mammals, the genetic variability may have two non-mutually exclusive contributions to developmental stability. First, heterozygosity creates increased biochemical diversity allowing for the buffering of dynamic environmental perturbations, thus stabilizing developmental trajectories [29]. Second, heterozygosity increases mother–fetus genetic distance, especially with respect to major histocompatibility complex (MHC) genes. The MHC genes are implicated in the immune dialogue between the mother and fetus, regulating embryonic implantation and growth [30,31]. Increased mother–fetus MHC distance is generally favorable for embryonic growth and the fitness of the offspring [32,33,34]. Thus, we wondered whether mother–fetus MHC-mediated immune cross-talk would impact the developmental/transcriptional stability of C57BL/6J inbred mice developing in surrogate mothers with identical or distinct MHC haplotypes.

To address this question, we first established a statistical framework to dissect the “extrinsic” and “intrinsic” components of gene noise using PCA projection, followed by GAMLSS modeling of eigenvalues. Next, we showed that the transfer of two-cell embryos from the C57BL/6J inbred mouse strain to surrogate mothers with different MHC haplotypes (1) decreased “extrinsic” and “intrinsic” (FA) gene expression noise in the forelimbs of embryos on day 16.5 of pregnancy, (2) had a positive impact on embryonic mass, and (3) stabilized fluctuations in embryonic growth. The control over embryonic growth and its stability could be mediated by the placental growth factor (PLGF), a critical factor stimulating embryonic development by promoting placental vasculo-angiogenesis [35,36]. PLGF was positively associated with embryonic mass, and fluctuations in PLGF concentrations were decreased in the placentas of BALB/c surrogate mothers carrying C57BL/6J pups. Interestingly, the “intrinsic” gene noise was negatively correlated with embryonic mass and PLGF concentrations, but it was positively associated with noise in these traits. Thus, we propose that the MHC-mediated mother–fetus immune cross-talk coordinates transcriptional and developmental stability resulting in improved embryonic growth and, therefore, fitness.

## 2. Results

### 2.1. Estimation of “Extrinsic”/“Intrinsic” Noise from the Log-Transformed Gene/Trait Expressions

Let us define gene expression as a log-transformed mRNA copy number (w=log(g)), and note that the variance of w is approximately equal to the squared coefficient of variation of g: $\sigma_{w}^{2}\approx \sigma_{g}^{2}/\mu_{g}^{2}=\eta_{g}^{2}$ (μ—mean), often referred to as noise [5,7]. This first-order Taylor approximation holds well for $\eta_{g}^{2}<1$. Otherwise, if g is a lognormal random variable defining mRNA concentration, a natural distribution for gene expression [37], $\sigma_{w}^{2}=log\left(1+\eta_{g}^{2}\right)$, but this makes further derivations unnecessarily cumbersome. Then, considering that g is conditioned on upstream drive ξ, and g and w are evaluated on the left (u=log(l)) and right (v=log(r)) sides, from the Equations (1) and (4), the variance of w partitions as:

(5) $$\sigma_{w}^{2}\approx \eta_{g}^{2}=\frac{\sigma_{g}^{2}}{\mu_{g}^{2}}=\underset{“\mathrm{extrinsic}”}{\underbrace{\mathrm{Var}[E(g|\;\xi )]/\mu_{g}^{2}}}+\underset{“\mathrm{intrinsic}”}{\underbrace{E\left[\mathrm{Var}\left(g|\;\xi \right)\right]/\mu_{g}^{2}}}\approx \frac{1}{4}\underset{“\mathrm{extrinsic}”}{\underbrace{\sigma_{l+r}^{2}/\mu_{g}^{2}}}+\frac{1}{4}\underset{“\mathrm{intrinsic}”}{\underbrace{\sigma_{l-r}^{2}/\mu_{g}^{2}}}$$

$$\sigma_{w}^{2}\approx \frac{1}{4}\underset{“\mathrm{extrinsic}”}{\underbrace{\sigma_{u+v}^{2}}}+\frac{1}{4}\underset{“\mathrm{intrinsic}”}{\underbrace{\sigma_{u-v}^{2}}}.$$

From Equations (2) and (3),

(6) $$\sigma_{u+v}^{2}\approx \sigma_{l+r}^{2}/\mu_{g}^{2}\approx 2\frac{\sigma_{g}^{2}}{\mu_{g}^{2}}\left(1+\rho_{l,r}\right)=2\eta_{g}^{2}\left(1+\rho_{l,r}\right)=\eta_{ext.}^{2}$$

(7) $$\sigma_{u-v}^{2}\approx \sigma_{l-r}^{2}/\mu_{g}^{2}\approx 2\frac{\sigma_{g}^{2}}{\mu_{g}^{2}}\left(1-\rho_{l,r}\right)=2\eta_{g}^{2}\left(1-\rho_{l,r}\right)=\eta_{int.}^{2}$$

From this, it is clear that the variance of the log-transformed variable partitions into “extrinsic” and “intrinsic” components of the noise of the original variable:

(8) $$\sigma_{w}^{2}\approx \eta_{g}^{2}\approx \frac{1}{4}\underset{“\mathrm{extrinsic}”}{\underbrace{\eta_{ext.}^{2}}}+\frac{1}{4}\underset{“\mathrm{intrinsic}”}{\underbrace{\eta_{int.}^{2}}}.$$

Thus, measuring FA as $\mathrm{FA}=\sigma_{u-v}^{2}\approx \eta_{int.}^{2}$ for the log-transformed gene expression values leads to an estimation of the “intrinsic” gene noise in the mRNA copy number. It is important to draw such a relationship between the variances of untransformed and often used log-transformed variables to avoid confusion regarding different gene/trait fluctuation measures, i.e., variance and the coefficient of variation (noise).

Note that $\mathrm{FA}=\sigma_{u-v}^{2}\approx \eta_{int.}^{2}$ is invariant to directional additive asymmetry, unlike other measures of FA, such as the mean absolute deviation. Indeed, if values for one side are systematically shifted relative to the other by a constant, e.g., u~a+v; then $FA=\mathrm{Var}\left[u-v-a\right]=\sigma_{u-v}^{2}\approx \eta_{int.}^{2}$. The same is true for $\sigma_{u+v}^{2}\approx \eta_{ext.}^{2}$. This makes $\sigma_{u-v}^{2}$ and $\sigma_{u+v}^{2}$ robust measures of the “intrinsic” and “extrinsic” gene noise, invariant to normalization. Indeed, if we express u and v as library-sized normalized mRNA counts: $u^{*}=\log\left(l/n_{l}\right)$ and $v^{*}=\log\left(r/n_{r}\right)$; then, $\sigma_{u^{*}\pm v^{*}}^{2}=\;\mathrm{Var}\left[u\pm v-\left(\log\left(n_{l}\right)\pm \log\left(n_{r}\right)\right)\right]=\sigma_{u\pm v}^{2}$.

However, in the presence of directional multiplicative asymmetry (u~av), $\sigma_{u\pm av}^{2}\neq \;\sigma_{u\pm v}^{2}$, as $\sigma_{u}^{2}=a^{2}\sigma_{v}^{2}$.

To eliminate such asymmetry, it is sufficient to perform a PCA. From the orthogonal projection of matrix w=(u,v) onto eigenvectors $\left({\vec{v}}_{1},{\vec{v}}_{2}\right)$, one can find eigenvalues $\lambda_{1}=\sigma_{PC1}^{2}$ and $\lambda_{2}=\sigma_{PC2}^{2}$. The $\lambda_{1}$ and $\lambda_{2}$ correspond to the “extrinsic” and “intrinsic” decomposition of gene noise. Indeed, for a special case a≈1 and $\sigma_{u}^{2}\approx \sigma_{v}^{2}$,

(9) $$\lambda_{1}=\sigma_{PC1}^{2}\approx \frac{1}{2}\sigma_{\left(u+v\right)}^{2}\approx \frac{1}{2}\eta_{ext.}^{2}$$

and

$$\lambda_{2}=\sigma_{PC2}^{2}\approx \frac{1}{2}\sigma_{\left(u-v\right)}^{2}\approx \frac{1}{2}\eta_{int.}^{2}$$

When a differs significantly from 1, the exact solutions for $\lambda_{1}$ and $\lambda_{2}$ become cumbersome, but the geometric interpretation of eigenvalues and their correspondence to the “extrinsic” and “intrinsic” noise components remains the same. Thus, in the further analysis, we used PCA transformation to cancel the directional multiplicative asymmetry and expressed fluctuating asymmetry/“intrinsic” gene noise through $\lambda_{2}=\sigma_{PC2}^{2}$, as

(10) $$\mathrm{FA}=\sigma_{\left(u-v\right)}^{2}\approx \eta_{int.}^{2}\approx 2\lambda_{2}$$

It is obvious that all the above could be applied to any trait.

### 2.2. Estimation of Pulled “Extrinsic”/“Intrinsic” Noise

An assessment of “intrinsic” noise for an individual requires multiple traits. Denote $W=\left\{w_{1},\ldots ,w_{k}\right\}$ as log-transformed gene/trait expression values for a set of genes/traits $\left\{w_{1},\ldots ,w_{k}\right\}$. W is conditioned (a) on the expression values of each gene/trait w, and, for simplicity, (b) on a common upstream drive ζ. Again, if W is evaluated for the left ($U=\left\{u_{1},\ldots ,u_{k}\right\}$) and right ($V=\left\{v_{1},\ldots ,v_{k}\right\}$) sides, then

(11) $$\sigma_{W}^{2}=\mathrm{Var}\left[E\left(W|w,\zeta \right)\right]+E\left[\mathrm{Var}\left(W|\;w,\zeta \right)\right]\approx \frac{1}{4}\sigma_{U+V}^{2}+\frac{1}{4}\sigma_{U-V}^{2}$$

The conditioning of W on w can be eliminated by taking the mean-centered values for each gene: $W^{*}=\left\{w_{1}-\mu_{w_{1}},\ldots ,w_{k}-\mu_{w_{k}}\right\}$, and

(12) $$\sigma_{W^{*}}^{2}=\underset{“\mathrm{extrinsic}”}{\underbrace{\mathrm{Var}\left[E\left(W^{*}|\zeta \right)\right]}}+\underset{“\mathrm{intrinsic}”}{\underbrace{E\left[\mathrm{Var}\left(W^{*}|\zeta \right)\right]}}\approx \frac{1}{4}\underset{“\mathrm{extrinsic}”}{\underbrace{\sigma_{U^{*}+V^{*}}^{2}}}+\frac{1}{4}\underset{“\mathrm{intrinsic}”}{\underbrace{\sigma_{U^{*}-V^{*}}^{2}}}\approx \frac{1}{4}\eta_{ext.}^{2}+\frac{1}{4}\eta_{int.}^{2}$$

Thus, pulled “extrinsic” and “intrinsic” noise components can be estimated for all genes/traits by calculating variances of the log-transformed mean-centered gene/trait expression sums $\sigma_{U^{*}+V^{*}}^{2}$ and differences $\sigma_{U^{*}-V^{*}}^{2}$ for the left and right sides. For gene expression, a common upstream drive ζ can be represented by global modulations in chromatin structure [38] caused, for example, by changes in temperature, intracellular pH, and other environmental/metabolic stimuli [39,40].

In practical terms, the estimation of the pulled noise components only requires the PCA transformation of the matrix of the left/right log-transformed, mean-centered gene/trait expressions ($W^{*}=\left(U^{*},V^{*}\right)$) to ensure the elimination of a potential directional multiplicative asymmetry. Then, the first eigenvalue ($\Lambda_{1}^{*}$) would correspond to $\eta_{ext.}^{2}$, and the first ($\Lambda_{2}^{*}$)—to $\eta_{int.}^{2}$:

(13) $$\Lambda_{1}^{*}\approx \frac{1}{2}\sigma_{U^{*}+V^{*}}^{2}\approx \frac{1}{2}\eta_{ext.}^{2}$$

$$\Lambda_{2}^{*}\approx \frac{1}{2}\sigma_{U^{*}-V^{*}}^{2}\approx \frac{1}{2}\eta_{int.}^{2}$$

assuming that $\sigma_{U^{*}}^{2}\approx \sigma_{V^{*}}^{2}$. It can also be noted that $\eta_{int.}^{2}$ could be found directly from the original non-centered left/right gene/trait expression matrix W=(U,V), following a PCA transformation. Indeed, $\sigma_{U^{*}-V^{*}}^{2}=Var\left[\ldots ,\left(u_{i}-\mu_{w_{i}}\right)-\left(v_{i}-\mu_{w_{i}}\right),\ldots \right]=Var\left[\ldots ,u_{i}-v_{i},\ldots \right]=\sigma_{U-V}^{2}$, and

(14) $$\Lambda_{2}\approx \frac{1}{2}\sigma_{U-V}^{2}=\frac{1}{2}\sigma_{U^{*}-V^{*}}^{2}\approx \frac{1}{2}\eta_{int.}^{2}$$

### 2.3. Estimation of Pulled “Intrinsic” Gene Noise from FA for A Subset of Genes and the Formation of the Distal–Proximal “Intrinsic” Gene Noise Gradient in Mouse Embryo Epiblast

**Figure 1:**
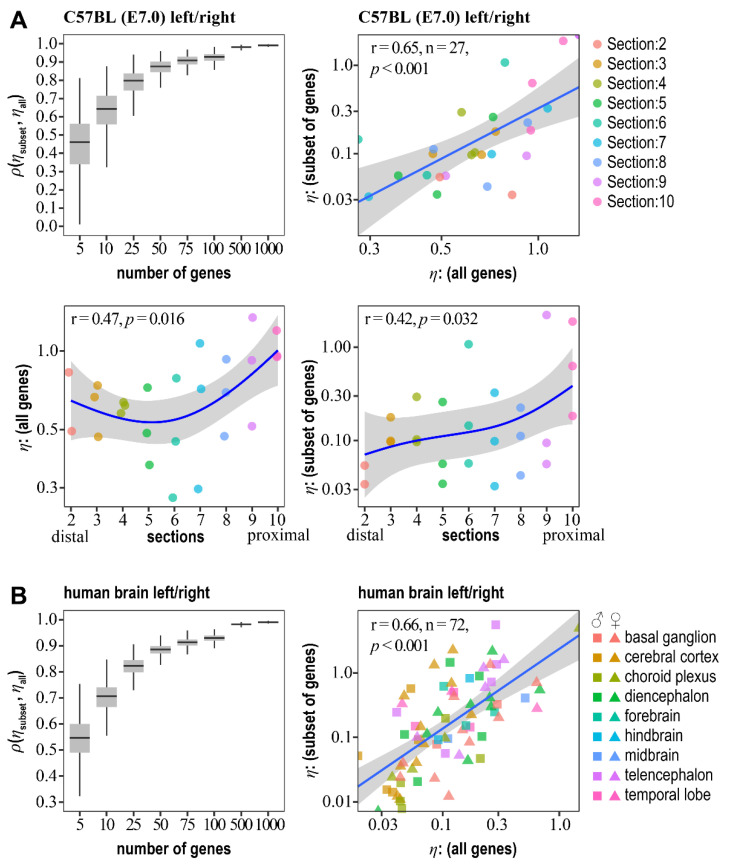
The distal–proximal gradient of pulled “intrinsic” gene noise in mouse embryo epiblast and correlations between pulled “intrinsic” gene noise estimated from the entire transcriptomes and random subsets of genes. (**A**) Boxplots of correlations of pulled “intrinsic” transcriptome noise ($\eta_{all}$) for mouse embryo epiblast left/right sections with estimates calculated for random subsets of genes ($\eta_{subset}$) (top-left panel). Correlation between pulled “intrinsic” gene noise estimates from the entire left/right transcriptomes and a selected subset of genes used for further RT-qPCR analysis (Supplementary Table S1) (top-right panel). Bottom panels: the distal–proximal gradient of pulled “intrinsic” transcriptome noise in mouse embryo epiblast. The $\eta_{all}$ (left panel) and $\eta_{subset}$ (right panel) were estimated for the entire transcriptome and selected for this study’s subset of genes (Table S1), respectively. The blue curve indicates the fitted P-spline model and grey ribbon—95% confidence interval. (**B**) Correlations between pulled “intrinsic” gene noise estimated from the entire transcriptomes and subsets of genes for human embryos’ left/right brain structures.

Although RNA-seq is widely adopted in studies of the dynamics of transcriptomic landscapes, RT-qPCR remains the method of choice for diagnostics. Thus, we wondered whether the pulled “intrinsic” gene noise for the entire transcriptional landscape could be estimated from a subset of genes for an individual. To this end, we used gene expressions measured on the left/right sides of the epiblast of C57BL/6 embryos at stage E7.0 [41]. The RNA-seq data (GSE65924) represented three embryos sliced into 11 segments (for the analysis, we used the data from 9 slices) along the anterior–posterior axis, thus yielding 27 individual, paired samples. We also used RNA-seq data (E-MTAB-4840) for the left/right paired human embryo brain transcriptomes of the 8–14-weeks-post-conception embryos [42]. In total, 72 paired human embryo samples from different brain structures were used for the analysis. The mRNA counts were normalized as FPKM (fragments per kilobase per million) and log-transformed. A further analysis was performed for genes with non-zero expression across all the samples (~9500 genes for mice and ~22,900 for humans). Following PCA for each paired sample, we evaluated pulled “intrinsic” gene noise as $\eta_{int.}^{\left[all\right]}\approx \sqrt{2\Lambda_{2}}$ (see Equation (14)). Then, we formed 1000 subsets, each consisting of 5, 10, 25, 50, 75, 100, 500, and 1000 randomly selected genes, and estimated the pulled “intrinsic” gene noise for each subset. We noted a significant correlation between $\eta_{int.}^{\left[all\right]}$ and $\eta_{int.}^{\left[subset\right]}$, even for the subsets consisting of five genes (Figure 1). Starting from ≥10 genes, the median correlation of pulled “intrinsic” gene noise evaluated on all genes and random subsets was ≳0.65 for mice and ≳0.7 for humans (Figure 1). It quickly approximated 1, starting from 100 genes (Figure 1). Thus, we concluded that the pulled “intrinsic” noise of the entire transcriptional landscape could be deduced from a small subset of genes (≥10) with good to very high accuracy.

Next, we noted a significant increase in the pulled “intrinsic” gene noise from the distal to proximal parts of the mouse embryo epiblast (Figure 1A). This trend was the same for the pulled “intrinsic” gene noise estimated genome-wide and from a small diagnostic subset of genes used for further analysis (Figure 1A, Table S1). The proximal epiblast is in the direct vicinity of the ectoplacental cone, and, in eutherians, the placenta is the sole communication interface between the mother and her fetus. Thus, the distal–proximal “intrinsic” gene noise gradient formation indicates the role of mother–fetus interaction in controlling gene expression stability.

### 2.4. A Model of Mother–Fetus MHC-Mediated Immune Cross-Talk

To model various MHC-mediated mother–fetus immune interactions, two-cell embryos of the C57BL/6J inbred strain were transferred to surrogate mothers of (a) the same H^2b^ MHC haplotype of the C57BL/6J strain, (b) the H^2g7^ MHC haplotype of the NOD-SCID immunodeficient strain, and (c) the H^2d^ MHC haplotype of the BALB/c strain. Thus, the first group models represents syngeneic H^2b^-H^2b^ mother–fetus cross-talk, the second represents allogeneic H^2g7^-H^2b^ mother–fetus interaction under severe combined immunodeficiency [43], and the third explores immunocompetent allogeneic H^2d^-H^2b^ mother–fetus communication.

The mean number of implanted and live C57BL/6J embryos did not differ significantly between surrogate mother groups (Table S2). The immunodeficient NOD-SCID dams had higher pre-implantation and total embryonic losses than the females from the immunocompetent groups C57BL/6J and BALB/c (Figure S1A). One of the indicators of a mother’s immune response to different MHC combinations between the mother and fetus is a change in the mass of immunocompetent organs (thymus and spleen). The thymus mass did not change in any of the groups on day 16.5 of pregnancy. On the contrary, the spleen mass increased significantly in the NOD-SCID and BALB/c but not in the C57BL/6J surrogate mothers, suggesting the initiation of mother–fetus immune cross-talk caused by allogeneic MHC combinations (Figure S1B). Expectedly, the thymus and spleen masses were lower in the NOD-SCID immunodeficient surrogate mothers (Figure S1B), which could partially impair the mother–fetus immune interaction.

The C57BL/6J embryonic growth, assessed by the mass of E16.5 embryos, was positively stimulated by the allogeneic MHC combination in the BALB/c immunocompetent surrogate mothers. The C57BL/6J embryos developed in the same MHC haplotype surrogate mothers had the lowest mass on day 16.5 of pregnancy. In the immunodeficient NOD-SCID dams, the growth of C57BL/6J embryos was intermediate compared to the C57BL/6J and BALB/c surrogate mothers (Figure S1C). The masses of the placentas and fetoplacental indices (FP) varied between groups. The FP were highest for the BALB/c immunocompetent surrogate mothers and the lowest for the immunodeficient NOD-SCID dams. The maximum placental weight was recorded when the C57BL/6J embryos were transferred to immunodeficient NOD SCID females (Figure S1C). Thus, the allogeneic MHC mother–fetus immune cross-talk positively modulates embryonic growth in immunocompetent surrogate mothers.

### 2.5. Mother–Fetus Immune Cross-Talk Modulates “Extrinsic”/“Intrinsic” Gene Noise

**Figure 2:**
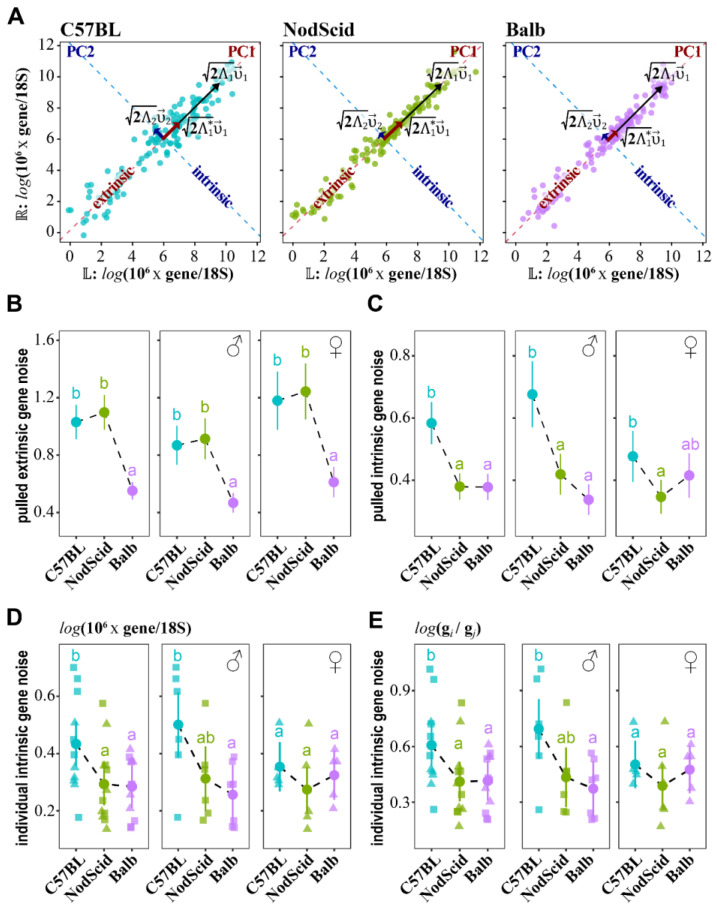
The impact of mother–fetus MHC-mediated immunogenic cross-talk on the “extrinsic”/“intrinsic” gene noise in C57BL/6J embryos. (**A**) PCA decomposition of 18S rRNA-normalized-log-transformed gene expressions in the left (L) and right (ℝ) forelimbs of C57BL/6J embryos on day 16.5 of gestation. The embryos were carried by C57BL/6J (*n* = 11 embryos carried by m = 8 females), NOD-SCID (*n* = 12, m = 8), and BALB/c (*n* = 12, m = 7) surrogate mothers. The PC1 and PC2 axis and eigenvectors $\sqrt{2\Lambda_{1}}{\vec{v}}_{1}$, $\sqrt{2\Lambda_{2}}{\vec{v}}_{2}$, and $\sqrt{2\Lambda_{1}^{*}}{\vec{v}}_{1}$ are indicated, where $\Lambda_{1}^{*}$ corresponds to the 1st eigenvalue of a gene-wise, mean-centered left/right expression matrix. (**B,C**) Mother–embryo interaction has an impact on pulled “extrinsic” ($\eta_{ext.}\approx \sqrt{2\Lambda_{1}^{*}}$) (**B**) and “intrinsic” ($\eta_{int.}\approx \sqrt{2\Lambda_{2}}$) (**C**) gene expression noise estimated group-wise for C57BL/6J embryos developing in C57BL/6J, NOD-SCID, and BALB/c surrogate mothers. The estimates were determined for either each mother’s genotype or separately for each mother’s genotype and fetus sex: C57BL/6J (*n*♂ = 6, *n*♀ = 5), NOD SCID (*n*♂ = 6, *n*♀ = 6), and BALB/c (*n*♂ = 5, *n*♀ = 7). Circles correspond to GAMLSS model-estimated coefficients: (**B**) $\sqrt{2}{\hat{\sigma }}_{PC1}\approx {\hat{\eta }}_{ext.}$ and (**C**) $\sqrt{2}{\hat{\sigma }}_{PC2}\approx {\hat{\eta }}_{int.}$, and whiskers denote 95% confidence intervals. Letters indicate significant between-group differences at FDR < 0.05 for multiple comparisons of the model-estimated coefficients by Wald tests. (**D**,**E**) Estimations of pulled “intrinsic” gene noise ($\sqrt{2\Lambda_{2}}\approx \sqrt{2}\sigma_{U-V}\approx \eta_{int.}$, Equation (14)) for each embryo using left (U)/right (V) 18S rRNA-normalized gene expressions (**D**) or ΔCts of all pair-wise gene combinations (**E**). Squares (♂) and triangles (♀) indicate individual values of pulled “intrinsic” gene noise, circles—mean values for each surrogate mother group, and whiskers—95% confidence intervals. Letters indicate multiple *t*-test comparisons with significant differences at FDR < 0.05.

To estimate the impact of MHC-mediated mother–fetus immune cross-talk on pulled gene expression stability, we have semi-randomly selected 13 genes (Supplementary Table S1). For the selected genes, (a) the mean expressions did not differ between experimental surrogate mother groups (Figure S2A), and (b) the pulled “intrinsic” gene subset noise estimates correlated well with entire transcriptome (Figure 1). The expressions were determined on day 16.5 of gestation in the left/right forelimbs by RT-qPCR. The Ct values for each gene were normalized to the expression of 18S rRNA as $\Delta \mathrm{Ct}={\mathrm{Ct}}_{g}-{\mathrm{Ct}}_{18S}=log_{2}\left(g/18S\right)$ yielding log-transformed estimates of the mRNA copy numbers. For each MHC surrogate mother haplotype group, the ΔCt expression matrices (W=(U,V)) containing left (U)/right (V) gene expressions for each embryo were constructed, and $\left({\vec{v}}_{1},{\vec{v}}_{2}\right)$ eigenvectors were found (Figure 2A). Then, ΔCt expression matrices were gene-wise mean-centered $W^{*}=\left(U^{*},V^{*}\right)$ and projected to $\left({\vec{v}}_{1},{\vec{v}}_{2}\right)$, resulting in the decomposition of $W^{*}$ into PC1/PC2 scores. To estimate the group-wise effects of the surrogate mothers’ MHC haplotype on the pulled “extrinsic”/“intrinsic” gene noise of the developing embryos, we performed distributional modeling of the PC1 and PC2 variances with a GAMLSS (Generalized Additive Models for Location, Scale, and Shape) approach [7,44]:

(15) $$\sigma_{PC1}\sim {\mathrm{MHC}}_{\left[i\right]}{\hat{\sigma }}_{PC1\left[i\right]}$$

$$\sigma_{PC2}\sim {\mathrm{MHC}}_{\left[i\right]}{\hat{\sigma }}_{PC2\left[i\right]}$$

where ${\mathrm{MHC}}_{\left[i\right]}$ is a surrogate mother group. Note that the group-wise effects on the means of PC1 and PC2 ($\mu_{PC1}$ and $\mu_{PC2}$) were modeled simultaneously for the correct estimation of the ${\hat{\sigma }}_{PC1}$ and ${\hat{\sigma }}_{PC2}$. The GAMLSS-evaluated parameters ${\hat{\sigma }}_{PC1}\pm se\left({\hat{\sigma }}_{PC1}\right)$ and ${\hat{\sigma }}_{PC2}\pm se\left({\hat{\sigma }}_{PC2}\right)$ yielded group-wise estimations of the “extrinsic” (${\hat{\eta }}_{ext.}\approx \sqrt{2}{\hat{\sigma }}_{PC1}=\sqrt{2{\hat{\Lambda }}_{1}^{*}}$) and “intrinsic” (${\hat{\eta }}_{int.}\approx \sqrt{2}{\hat{\sigma }}_{PC2}=\sqrt{2{\hat{\Lambda }}_{2}^{}}$) components of gene noise, respectively (Equations (13) and (14)), and were compared between groups by Wald tests (Figure 2B,C).

This analysis revealed a significant reduction in the pulled “extrinsic” gene noise on a group-wise level for the C57BL/6J embryos gestated in BALB/c surrogate mothers compared to C57BL/6J and NOD-SCID (Figure 2B). The effect was equally significant for both male and female embryos. The embryo sex was determined by the SRY marker gene [45]. The pulled “intrinsic” gene noise was reduced in the groups of embryos developing in allogeneic NOD-SCID and BALB/c surrogate mothers compared to syngeneic C57BL/6J dams (Figure 2C). This effect was most pronounced for male embryos (Figure 2C). Similar results were obtained from estimating the “extrinsic”/“intrinsic” noise for each studied gene using the eq. [9] (Figure S2B). Thus, we conclude that the increased MHC distance between the mother and fetus is favorable for stabilizing the expression of selected genes and, possibly, of the entire transcriptome (Figure 1) in developing embryos. This stabilization effect is better revealed on both the “extrinsic” and “intrinsic” levels in the male C57BL/6J embryos undergoing development in the allogeneic immunocompetent BALB/c surrogate mothers.

Next, we estimated the pulled “intrinsic” gene noise for each embryo. To this end, we applied PCA transformations to individual ΔCt expression matrices and, using eq. [14], calculated the “intrinsic” gene noise (Figure 2D). For the diagnostics of an individual’s transcriptional stability, using a single “housekeeping” gene to normalize the RT-qPCR Ct values could slightly elevate the “intrinsic” gene noise estimates. Indeed, here, we used the expression of the 18S rRNA gene for normalization, which is in itself a random variable. Thus, we also evaluated the “intrinsic” gene noise from ΔCts of all the pair-wise gene combinations (Figure 2E). The two measurements were highly correlated (r = 0.99), and both measures of an individual’s “intrinsic” gene noise substantiated our conclusion that immunocompetent allogeneic H^2d^-H^2b^ mother–fetus cross-talk reduces intra-individual gene expression fluctuations in male embryos (Figure 2D,E).

### 2.6. Association of “Intrinsic” Gene Noise with Embryo Mass and Its Phenotypic Stability

**Figure 3:**
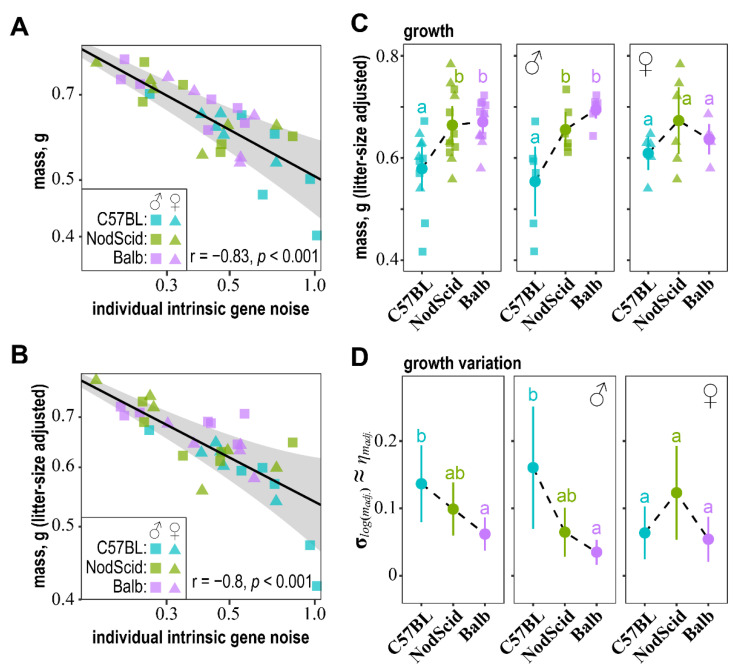
Relationships between embryos pulled “intrinsic” gene noise, their mass, and phenotypic noise. (**A,B**) GAMLSS models of the relations of non-adjusted (**A**) and litter-size-adjusted (**B**) C57BL/6J embryonic mass (log(m)) with individual estimates of the pulled “intrinsic” gene noise ($\eta_{int.}$, Figure 2D). Grey ribbons indicate the model-estimated 25–75 centile region for the normally-distributed log(m). Correlation coefficients are indicated in the bottom-right corner; the GAMLSS model coefficients are given in Table S3. Squares indicate male embryos, and triangles represent female embryos. Surrogate mother groups C57BL/6J, NOD-SCID, and BALB/c are color-coded. (**C**) Litter-size-adjusted mass of C57BL/6J embryos developed in C57BL/6J, NOD-SCID, and BALB/c surrogate mothers on day 16.5 of pregnancy, for which gene noise (Figure 2) and sex (squares—♂, triangles—♀) were determined. Circles indicate means for each surrogate mother group, whiskers—95% confidence intervals, and letters—multiple *t*-test comparisons with significant differences at FDR < 0.05. (**D**) GAMLSS estimation of the effect of surrogate mothers’ MHC haplotype on the noise of litter-size-adjusted-log-transformed embryonic mass ($\sigma_{log\left(m_{adj.}\right)}\approx \eta_{m_{adj.}}$). The two parameters (μ, σ) were fitted simultaneously: (1) $\mu_{log\left(m_{adj.}\right)}\sim {\mathrm{MHC}}_{\left[i\right]}{\hat{\mu }}_{\left[i\right]}$ and (2) $\sigma_{log\left(m_{adj.}\right)}\sim {\mathrm{MHC}}_{\left[i\right]}{\hat{\sigma }}_{\left[i\right]}$, under the assumption that $log\left(m_{adj.}\right)$ is normally distributed. Circles correspond to the GAMLSS model estimated ${\hat{\sigma }}_{log\left(m_{adj.}\right)}\approx {\hat{\eta }}_{m_{adj.}}$, whiskers correspond to 95% confidence intervals, and letters indicate significant between-group differences at FDR < 0.05.

Having established the impact of MHC-mediated mother–fetus immune communication on embryos’ gene expression stability, we then wondered how transcriptional stability relates to developmental stability. First, we noted that the individual estimates of the pulled “intrinsic” gene noise for the C57BL/6J embryos (Figure 2D) negatively correlated with their non-adjusted and litter-size-adjusted masses across all surrogate mother groups (Figure 3A,B). Second, we applied GAMLSS to model the dependencies of the log-transformed C57BL/6J embryonic mass (log(m)) distribution parameters (μ, σ) on the individual estimates of pulled “intrinsic” gene noise:

(16) $$\mu_{log\left(m\right)}\sim \alpha_{0}+\alpha log\left(\eta_{int.}\right)$$

(17) $$\sigma_{log\left(m\right)}\approx \eta_{m}\sim \beta_{0}+\beta log\left(\eta_{int.}\right)$$

Note that if log(m) is normally distributed, then $\sigma_{log\left(m\right)}^{2}=log\left(1+\eta_{m}^{2}\right)\approx \eta_{m}^{2}$, for $\eta_{m}^{2}<1$. Estimations of $\hat{\alpha }$ for the non-adjusted and litter-size-adjusted embryonic masses confirmed a significant negative association between embryonic growth and pulled “intrinsic” gene noise for the combined and each surrogate mother groups’ embryos (Figure 3A,B; Table S3). In contrast, the estimations of $\hat{\beta }$ indicated that phenotypic noise in embryonic mass ($\sigma_{log\left(m\right)}\approx \eta_{m}$) was positively related to the pulled “intrinsic” gene noise (Figure 3A,B; Supplementary Table S3). This relation was most pronounced for the variability of the litter-adjusted embryonic mass (Supplementary Table S3). These results uncovered positive associations between embryonic mass, the phenotypic stability of embryonic growth, and the robustness of the transcriptional landscape.

Consistent with these findings, we noted a significant increase in both the non-adjusted and litter-size-adjusted mass of the C57BL/6J male embryos gestated in the NOD-SCID and BALB/c compared to the C57BL/6J surrogate mothers (Figure 3C, Supplementary Figure S3A). Note that in Supplementary Figure S1C, the embryonic mass is given for the entire litters; here, we used only a subset of embryos, for which gene expression noise was evaluated and sex was determined. The GAMLSS estimations of $\sigma_{log\left(m\right)}\approx \eta_{m}$ also identified a significant reduction in phenotypic noise in the adjusted embryonic mass for the male embryos developed in the BALB/c compared to C57BL/6J surrogate mothers (Figure 3D, Supplementary Figure S3B). The adjusted embryonic mass noise was intermediate for the male embryos developing in the NOD-SCID dams (Figure 3D). The effects of MHC were insignificant with respect to female embryonic mass and growth noise. Note that the impact of the surrogate mothers’ MHC haplotype on embryonic mass and its phenotypic stability was concordant with the estimates of “intrinsic” gene expression stability (Figure 2D,E).

### 2.7. Association of “Intrinsic” Gene Noise with PLGF Expression and PLGF Noise

Growth factors, such as VEGF (vascular endothelial growth factor) and PLGF (placental growth factor), promote embryonic growth by inducing vasculo-angiogenesis in the placenta. Both are expressed by mother cells (uNK—uterine natural killer cells) and embryo trophoblast cells, indicating a mechanism of embryonic growth control through mother–fetus communication [35,36]. Thus, we wondered how VEGF and PLGF are associated with embryonic growth and its developmental and transcriptional stability in our model of MHC-mediated mother–fetus cross-talk. To this end, we measured VEGF and PLGF levels in the placental homogenates of the C57BL/6J embryos subjected to gene noise analysis. We noted a significant correlation of PLGF concentrations with both non-adjusted (r = 0.7, *p* < 0.001) and litter-size-adjusted (r = 0.74, *p* < 0.001) embryonic mass. A positive association between PLGF concentrations and litter-size-adjusted embryonic mass was significant for all surrogate mother groups: C57BL/6J (r = 0.81, *p* = 0.002), NOD-SCID (r = 0.58, *p* = 0.046), and BALB/c (r = 0.71, *p* = 0.009). Correlations of PLGF with non-adjusted embryonic mass were also significant for the C57BL/6J (r = 0.89, *p* < 0.001) and BALB/c (r = 0.60, p = 0.038) surrogate mother groups, but fell short of statistical significance for the NOD-SCID (r = 0.54, *p* = 0.067) group. VEGF correlated with neither non-adjusted (r = −0.22, *p* = 0.2) nor litter-size-adjusted (r = −0.07, *p* = 0.7) embryonic mass. This result indicates a more prominent role of PLGF compared to VEGF in controlling embryonic growth in our model of surrogate pregnancies, and, for further analysis, we focused on PLGF.

**Figure 4:**
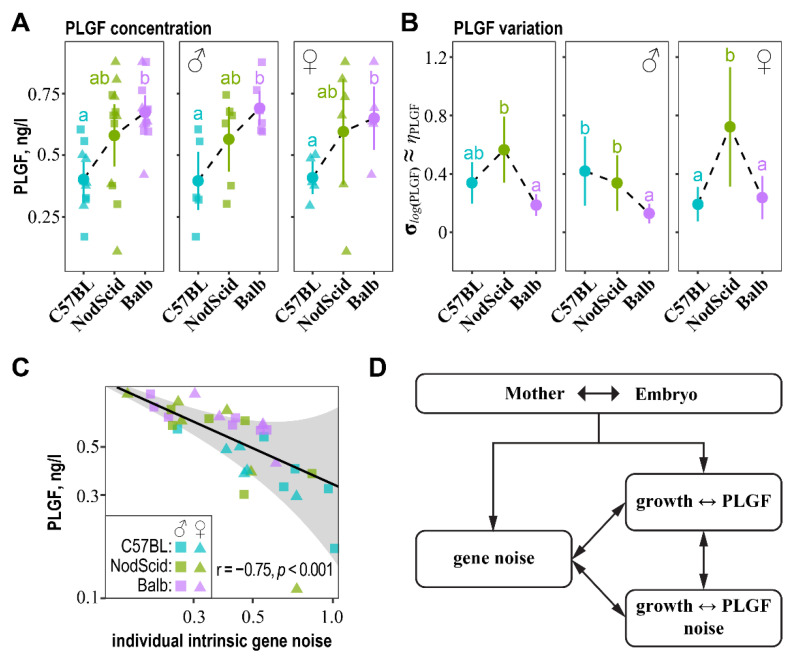
Relationships between PLGF, PLGF noise, and pulled “intrinsic” gene noise. (**A**) PLGF concentrations in placentas of C57BL/6J embryos developed in C57BL/6J, NOD-SCID, and BALB/c surrogate mothers on day 16.5 of pregnancy. Circles indicate means, whiskers—95% confidence intervals, and letters—significant differences at FDR < 0.05. (**B**) GAMLSS estimations of noise in PLGF concentrations. Circles indicate ${\hat{\sigma }}_{log\left(PLGF\right)}\approx {\hat{\eta }}_{PLGF}$, whiskers—95% confidence intervals, and letters—significant differences at FDR < 0.05. (**C**) GAMLSS model of the relations of placental PLGF (log(PLGF)) with individual estimates of the pulled “intrinsic” gene noise ($\eta_{int.}$). Grey ribbon indicates a 25–75 centile region. Correlation is shown in the bottom-right corner; the model coefficients are given in Table S4. (**D**) A model depicting the cross-talk between transcriptional stability (gene noise), PLGF levels, embryonic growth, and fluctuations in PLGF and embryonic growth mediated by mother–fetus MHC immune interactions (see main text for details).

The levels of PLGF were significantly higher in the placentas of the C57BL/6J embryos developing in the BALB/c surrogate mothers compared to C57BL/6J, and they were intermediate in the placentas of the embryos gestated in NOD-SCID (Figure 4A). The noise estimated by the GAMLSS model for the PLGF concentrations ($\sigma_{log\left(PLGF\right)}\approx \eta_{PLGF}$) was overall decreased in the placentas of embryos gestating in the BALB/c surrogate mothers compared to NOD-SCID, and, for the C57BL/6J surrogate mothers, it was intermediate (Figure 4B). The GAMLSS model of placental PLGF concentrations and noise revealed a significant association of both estimated parameters with pulled “intrinsic” gene noise. Increased transcriptional noise in the individual embryos was inversely related to PLGF levels but proportional to PLGF inter-individual noise (Figure 4C, Table S4). This result perfectly mirrors the relations between individual estimates of pulled “intrinsic” gene noise and embryonic growth, as well as the phenotypic stability of the inter-individual fluctuations in mass (Figure 3A,B).

## 3. Discussion

FA is a simple and often used estimator of the DI of symmetric traits [1,2,3,4,11]. Correlation is one of the widely employed measures of FA [11,12], but it measures association, not fluctuation. This could result in misleading conclusions about changes in the trait’s FA [12], especially when the trait’s variance changes (Figure S4). The other standard estimate of FA is the mean absolute left–right deviation [11,12]. However, in statistical terms, the proper measure of a trait’s fluctuation is its variance or noise (coefficient of variation). In principle, any biological trait can be viewed as a random variable x conditioned on the control variable ξ—x| ξ, where ξ represents a composition of all upstream (environmental) drives (stimuli) acting on x. Then, from the law of total variance, the variance (DI) of a left/right evaluated trait x ($\sigma_{x}^{2}$) partitions into “extrinsic” ($\sigma_{l+r}^{2}$) and “intrinsic” ($\sigma_{l-r}^{2}$) components, each of which depends on ξ (Equations (1)–(4)). Likewise, the DI of log-transformed traits partitions into noise: $\eta_{ext.}^{2}$ and $\eta_{int.}^{2}$(Equations (5)–(8)). Such partitioning highlights a distinction between the commonly discussed manifestations of the DI: the canalization (between-individual variability) and FA (inter-individual variability) [1,2,3,4]. Canalization corresponds to the “extrinsic” and FA to the “intrinsic” components of the DI.

Both components of DI can be readily estimated by projecting a matrix of the left/right measures of a trait x to eigenvectors. The 1st eigenvalue $\lambda_{1}=\sigma_{PC1}^{2}\approx \frac{1}{2}\sigma_{\left(l+r\right)}^{2}$ yields an estimation of the “extrinsic” and the 2nd eigenvalue $\lambda_{2}=\sigma_{PC2}^{2}\approx \frac{1}{2}\sigma_{\left(l-r\right)}^{2}$ of the “intrinsic” component of the DI ($\sigma_{x}^{2}$) (Equations (9)–(10)). The PCA-derived estimations of the “extrinsic” and “intrinsic” fluctuations are invariant to additive and/or multiplicative directional asymmetry. This analysis can be generalized to multiple traits (Equations (11)–(14)) and applied to evaluate transcriptional/developmental stability at an individual level. Further, using GAMLSS distribution parameter modeling [44], the fixed/random/mixed effects of categorical or continuous factors on the $\sigma_{PC1}^{2}$ and $\sigma_{PC2}^{2}$ can be estimated from the PC1 and PC2 scores. Thus, PCA in combination with GAMLSS provides a flexible framework for the statistical dissection of the “extrinsic” and “intrinsic” components of DI. Applying this framework, we investigated the impact of MHC-mediated mother–fetus immune interaction on transcriptional and developmental stability.

First, we established that the pulled genome-wide “intrinsic” gene noise could be estimated from a small random subset of genes for an individual with high accuracy (Figure 1). This argues for the presence of a global control variable ξ buffering the entire transcriptional landscape. Otherwise, if gene expression stability is primarily controlled on the local—as opposed to global—gene level, the effects of gene-specific upstream drives will probably cancel each other when the pulled “intrinsic” gene noise is evaluated from a random subset of genes. As a result, the observed correlations between the pulled “intrinsic” gene noise estimated for the entire transcriptome and random gene subsets would be unlikely (Figure 1).

In fact, there are several candidates for the global control variable ξ, which could relay information on environmental fluctuations to the entire chromatin structure, thus modulating genome-wide gene expression stability. For example, temperature modulates the stability of nucleosomes and, therefore, could exert a global control on gene noise and developmental stability [4,39,46,47]. Likewise, cellular stress, nutrients, aging, etc., induced changes in the intracellular pH affecting global histone acetylation, which, as a result, could interfere with genome-wide gene expression stability [40,48,49,50]. The placenta’s balance between extra- and intracellular pH and maternal–fetal thermoregulation are critical for embryonic growth and stable development [51,52].

We also propose that maternal signals transmitted through the placenta could orchestrate “intrinsic” gene noise in the developing embryo. Indeed, we noted the formation of the distal–proximal gradient in the E7.0 mouse embryo epiblast, where gene noise decreased in the proximal to the distal direction (Figure 1A). The proximal part is located in the vicinity of the placenta and, therefore, could be more exposed to maternal signals. As a result of placentally mediated mother-fetal communication, the spatial gradient of the pulled “intrinsic” gene noise could be formed.

Second, the evaluation of gene noise from a subset of diagnostic genes revealed the stabilization of the transcriptional landscape in the C57BL/6J embryos developing in the allogeneic NOD-SCID and BALB/c surrogate mothers compared to the syngeneic C57BL/6J surrogate dams. The reduction in pulled “intrinsic” gene noise was positively associated with growth (embryonic mass) and the phenotypic stability of the growth (variations in embryonic mass) across all the studied groups. The effects of the MHC-mediated mother–fetus cross-talk on the stabilization of gene expression and growth fluctuations were more pronounced in male than in female embryos. Such sexual dimorphism suggests a tighter control over transcriptional and phenotypic stability and, as a result, less sensitivity to MHC combinations in female progeny compared to male offspring. A greater sensitivity of male embryos to the syngeneic mother–fetus MHC combination manifested in the elevated gene expression, and the phenotypic noise could highlight a new potential mechanism for the “greater male variability hypothesis” [53,54,55]. As such, we propose that a reduced mother–fetus MHC distance drives greater transcriptional and, thus, growth phenotypic variability in males leading to a restriction of their growth (lower embryonic mass).

Finally, we found that the levels and expressional stability of PLGF, an embryonic growth-promoting factor [35,36], were positively associated with pulled “intrinsic” gene expression stability. Likewise, the levels and fluctuations in PLGF depended on the surrogate mother’s genotype, with the maximum concentrations and minimum noise attained in the placentas of the C57BL/6J male embryos developing in the BALB/c surrogate mothers.

Combining these findings (Figure 4D), we showed that MHC-mediated mother–fetus immune cross-talk directly impacts the overall gene expression noise, mean embryonic growth, and fluctuations in growth. Although it is challenging to explicate all the causalities, the stabilization of embryonic growth could be mediated by the stabilization of the PLGF levels caused by distant mother–fetus MHC combinations. The stabilization of PLGF could directly result from stabilizing the transcriptional landscape. As a result, transcriptional and developmental stability could positively impact embryonic growth and, thus, the fitness of offspring developed under fully established mother–fetus immune cross-talk.

## 4. Materials and Methods

### 4.1. Embryo Transfer

All experiments were performed at the Centre for Genetic Resources of Laboratory Animals, Institute of Cytology and Genetics of the Siberian Branch of the Russian Academy of Sciences (SB RAS). Mice were housed under specific pathogen-free (SPF) conditions in individually ventilated OptiMice cages (Animal Care Systems Inc., Centennial, CO, USA) in same-sex groups (5 per cage). An artificial photoperiod (14 h light: 10 h dark), adjustable temperature (22–24 °C), and humidity (40–50%) were maintained. All animals had access to water and granulated mouse food (SNIFF, Germany) ad libitum.

The three inbred mouse strains, namely, C57BL/6J, NOD-SCID, and BALB/c, were chosen for C57BL/6J embryo transfer experiments based on the known differences in the MHC haplotype. The two-cell embryo transfers were performed as previously described [32]. In brief, embryo donors, C57BL/6J females (8–10 weeks old), were superovulated by intraperitoneal administration of 7.5 IU of Folligon (MSD Animal Health, Pune, India) and, 48 h after, by injection with 7.5 IU of Chorulon (MSD Animal Health, Pune, India). The next day, two-cell C57BL/6J embryos were washed out from oviducts in M2 medium (Sigma-Aldrich, Burlington, MA, USA), collected into a drop of KSOM medium (Cosmo Bio, Tokyo, Japan) covered with mineral oil (Sigma-Aldrich, Burlington, MA, USA), and incubated at 37 °C under 5% CO_2_ until transfer to recipient females. The recipient females C57BL/6J, NOD-SCID, and BALB/c were mated with vasectomized males of the same strain and checked for vaginal plugs. The C57BL/6J two-cell embryos were transferred to pseudo-pregnant recipient females, namely, C57BL/6J (m = 12 females), NOD-SCID (m = 17), and BALB/c (m = 12), under gas anesthesia AErrane (Baxter Healthcare Corp., Deerfield, IL, USA).

### 4.2. Characteristics of Pregnancy, Embryo Growth, and PLGF and VEGF Immunoassays

Females were euthanized on day 16.5 of gestation and immunocompetent organs (thymus and spleen), fetuses, and placentas were weighted. Placentas, embryos’ tail tips, and left/right forelimbs were stored at −80 °C before the assays were applied.

The PLGF and VEGF concentrations were measured by PLGF ELISA and VEGF ELISA kits (Abcam, Fremont, CA, USA) in supernatants of placental homogenates. The assays were performed according to the manufacturer’s protocol. The sensitivity of the PLGF and VEGF assays were 0.526 pg/mL and 0.3 pg/mL, respectively. The intra- and inter-assays CV of the PLGF were 4.27% and 6.5%, respectively, and those of the VEGF were 5.6% and 7.9%, respectively.

Embryo sex was determined as described in [45]. In brief, a total of 500 μL of lysis buffer (100 mM NaCl; 10 mM Tris-HCl, pH 8; 25 mM EDTA, pH 8; 0.5% SDS, 1 μL proteinase K) was added to ~20 mg of mouse tails and incubated for 2 h at 55 °C. After incubation, samples were homogenized, incubated for an additional 15 min at 65 °C, centrifuged for 1 min at 10,000× *g*, and 50 μL of 3 M Sodium acetate was added to supernatants. PCR amplification of Y-chromosomal DNA that detects the male-determining gene SRY was used for sex determination [45].

### 4.3. Evaluation of Gene Expression by RT-qPCR

Isolation of RNA from left and right forelimbs of E16.5 fetuses was based on the guanidine thiocyanate-phenol-chloroform method. The samples (~100 mg) were homogenized, and RNA was extracted according to the manufacturer’s protocol (Biolabmix, Novosibirsk, Russia). The concentration of RNA was determined with NanoDrop 2000/2000c (ThermoFisher Scientific, Waltham, MA, USA). The cDNA was synthesized at 42 °C for 60 min using a random hexamer primer and 100 U of M-MLV reverse transcriptase according to the manufacturer’s instructions (Biolabmix, Novosibirsk, Russia). Briefly, 2 μL of RNA was incubated with 2 μM of random hexamer primer in 12 μL final reaction volume at 70 °C for 3 min and then cooled on ice. Then, 16 μL of the reaction mixture containing M-MLV reverse transcriptase was added, and the mixture was incubated at 25 °C—10 min, 42 °C—60 min, and 70 °C—10 min. The genes and primers used for RT-qPCR are listed in Table S1. An amount of 2 μL of cDNA samples was added to 20 μL of RT-qPCR SYBR Green master reaction mix containing 0.5 μM of primers. The amplification reaction was carried out on CFX96 PCR System (BioRad, Hercules, CA, USA). DNA was denatured for 5 min at 95 °C and then amplified in 45 cycles: 95 °C −15 s; 62 °C—50 s. All PCR products were checked by melting curve analysis.

RT-qPCR for each sample was duplicated. On average, the standard error of technical replicates expressed as ${\Delta \mathrm{Ct}}_{\mathrm{rep}}={\mathrm{Ct}}_{\mathrm{rep}1}-{\mathrm{Ct}}_{\mathrm{rep}2}$ was ${\hat{\sigma }}_{{\Delta \mathrm{Ct}}_{\mathrm{rep}}}=0.135\pm 0.0026$. This was negligible compared to the average left (U)/right (V) fluctuations of the normalized-to-18S-rRNA (18S) expressions of all measured genes ($U,V:\;\Delta \mathrm{Ct}={\mathrm{Ct}}_{g}-{\mathrm{Ct}}_{18S}$): ${\hat{\sigma }}_{U-V}=0.59\pm 0.017$ (*p* < 0.001, Walt test comparing ${\hat{\sigma }}_{{\Delta \mathrm{Ct}}_{\mathrm{rep}}}$ and ${\hat{\sigma }}_{U-V}$). Thus, for further analysis, we averaged technical replicates.

### 4.4. Statistical Analysis of Noise

The analysis of gene expression and phenotypic noise is detailed in the main text. In brief, for the estimation of “extrinsic” and “intrinsic” noise, the matrix containing the left (U) and right (V) ΔCt values ($\Delta \mathrm{Ct}={\mathrm{Ct}}_{g}-{\mathrm{Ct}}_{18S}$) was formed: W=(U,V). The eigenvectors $V=\left({\vec{v}}_{1},{\vec{v}}_{2}\right)$ of the covariance matrix Cov(U,V) were calculated with *R*’s *eigen* function (https://cran.r-project.org/). The gene-wise, mean-centered $W^{*}=\left(U^{*},V^{*}\right)$ was projected to eigenvectors: $PC1=W^{*}{\vec{v}}_{1}$, $PC2=W^{*}{\vec{v}}_{2}$. The eigenvalues ($\Lambda_{1}^{*}=\sigma_{PC1}^{2}$, $\Lambda_{2}^{*}=\Lambda_{2}=\sigma_{PC2}^{2}$) were estimated by GAMLSS using *R*’s *gamlss* package [7,17,44]. The estimated ${\hat{\sigma }}_{PC1}\approx \frac{1}{\sqrt{2}}{\hat{\eta }}_{ext.}$ and ${\hat{\sigma }}_{PC2}\approx \frac{1}{\sqrt{2}}{\hat{\eta }}_{int.}$ were compared between surrogate mother groups by the Wald test: $w=\frac{{\hat{\theta }}_{1}-{\hat{\theta }}_{2}}{\sqrt{{\left(se_{{\hat{\theta }}_{1}}\right)}^{2}+{\left(se_{{\hat{\theta }}_{2}}\right)}^{2}}}$ ($\hat{\theta }$—GAMLSS estimated parameter (${\hat{\sigma }}_{PC1}$, ${\hat{\sigma }}_{PC2}$, *etc.*); $se_{\hat{\theta }}$—standard error of the maximum likelihood estimate), which follows assymptotic z distribution. The *p*-values were FDR- (false discovery rate) adjusted for multiple comparisons. For more details on GAMLSS, refer to [44] and (https://www.gamlss.com/).

## Data Availability

The data supporting this study’s findings are available upon request.

## Supplementary Materials

The following supporting information can be downloaded at: https://www.mdpi.com/article/10.3390/ijms232012467/s1.
