# Supplementary material for: Mother–Fetus Immune Cross-Talk Coordinates “Extrinsic”/“Intrinsic” Embryo Gene Expression Noise and Growth Stability
Source: Int J Mol Sci. 2022 Oct 18;23(20):12467. doi: 10.3390/ijms232012467 (PMC9604428; doi:10.3390/ijms232012467)
Supplement: Supplementary file 1 [file ijms-23-12467-s001.zip › ijms-1906148-supplementary.pdf]

# Supplementary materials

## Mother–fetus immune cross-talk coordinates “extrinsic”/”intrinsic” embryo gene expression noise and growth stability

Tatyana Ivanovna Babochkina[3]; Ludmila Alekseevna Gerlinskaya[3]; Margarita Vladimirovna Anisimova[3]; Galina Vladimirovna Kontsevaya[3]; Natalia Aleksandrovna Feofanova[3]; Aliya Konstantinovna Stanova[3]; Mikhail Pavlovich Moshkin[2,3]; Yuri Mikhailovich Moshkin[1,3,*]

[1] Gene Learning Association, 1205 Geneva, Switzerland
[2] Department of Zoology and Animal Ecology, Tomsk State University, 634050 Tomsk, Russia
[3] Federal Research Center Institute of Cytology and Genetics, Siberian Branch of RAS, 630090 Novosibirsk, Russia
* Correspondence: yury.moshkin@gmail.com

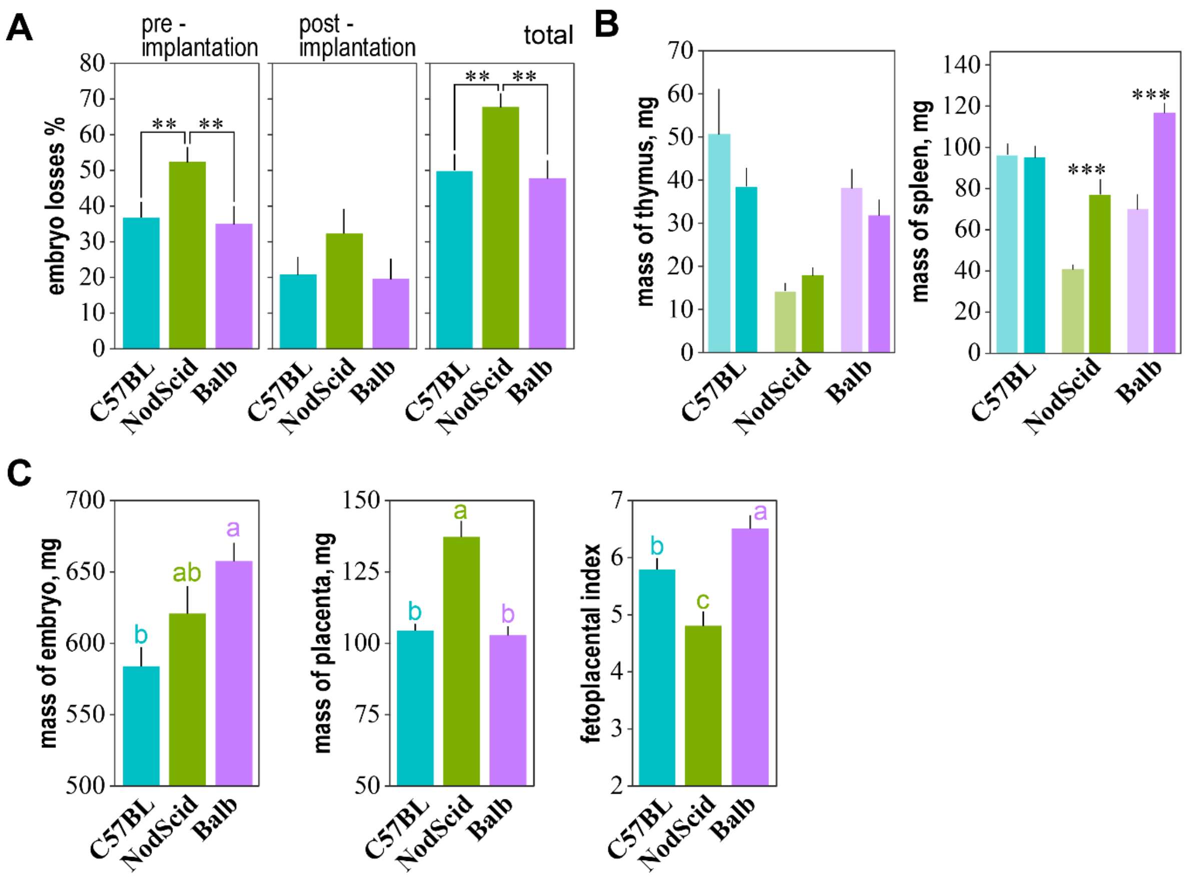


**Figure S1. Characteristics of C57BL/6J fetuses and surrogate mothers' immunocompetent organs response.**

**A)** Pre-, post-implantation, and total embryo losses in C57BL/6J, NOD-SCID, and BALB/c surrogate mothers. ** - Student's t-test, $p<0.01$.

**B)** Females' thymus and spleen mass in non-pregnant females (light-colored bars) and in pregnant (day 16.5 of surrogate pregnancy) females (dark-colored bars). *** - $p<0.001$, t-test; the number of non-pregnant/pregnant females: C57BL/6J (m = 3/13), NOD-SCID (m = 5/16) and BALB/c (m = 5/13).

**C)** C57BL/6J embryo weight, placenta weight, and fetoplacental index on day 16.5 of surrogate pregnancy. Letters indicate significant statistical differences at $p < 0.05$, LSD-test; the number of embryos by surrogate mother group: C57BL/6J (n = 61), NOD-SCID (n = 46) and BALB/c (n = 49).

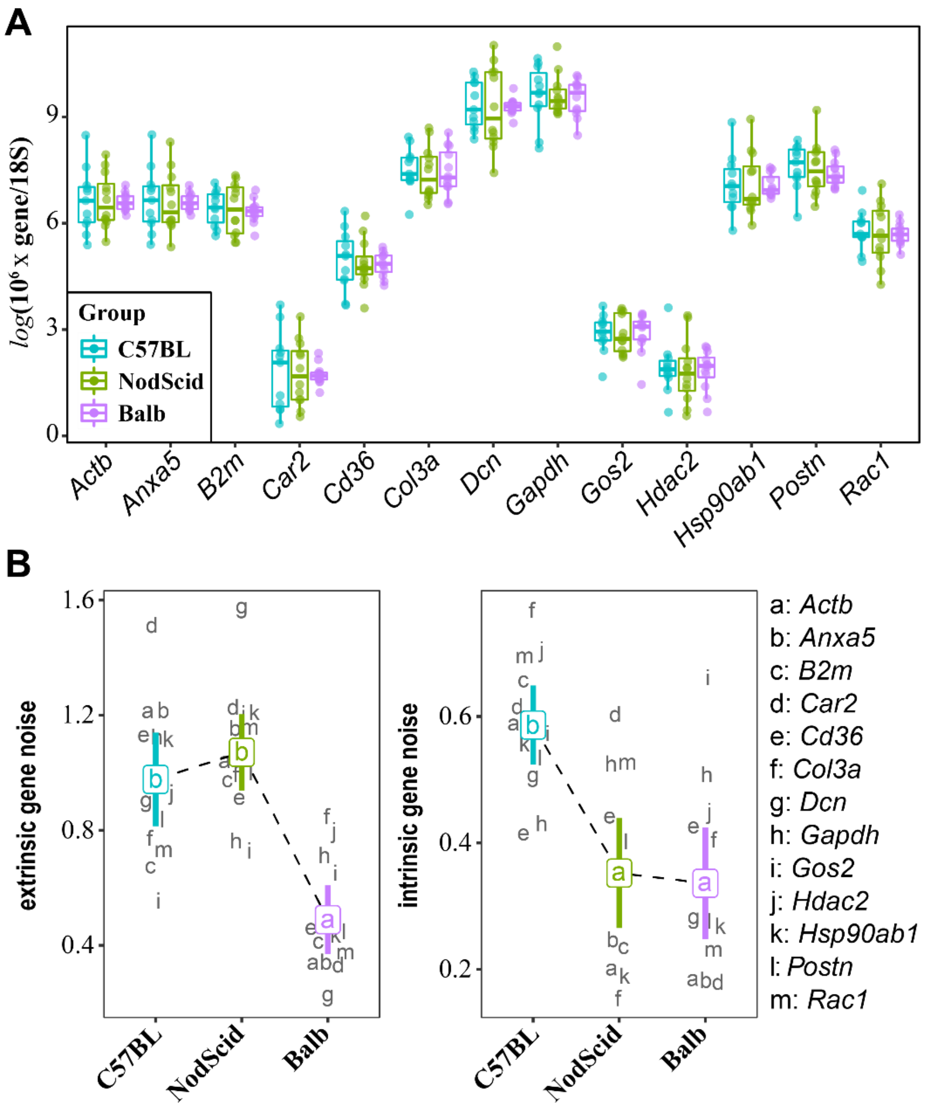


**Figure S2. The impact of mother-fetus MHC-mediated interactions on gene-wise "extrinsic"/"intrinsic" fluctuations in left/right forelimbs of C57BL/6J embryos.**

**A)** Left/right mean RT-qPCR ΔCt $= log(10^6\ g/18S)$ values for each gene measured on day 16.5 of gestation in forelimbs of C57BL/6J embryos, developing in C57BL/6J, NOD-SCID, and BALB/c surrogate mothers. ANOVA and pair-wise comparisons revealed no significant differences between surrogate mother groups' ΔCts of selected genes.

**B)** Estimations of "extrinsic" ($\eta_{ext.} \approx \sqrt{2\lambda_1}$) and "intrinsic" ($\eta_{int.} \approx \sqrt{2\lambda_2}$) gene noise for the selected genes following PCA decomposition of left/right gene expression (ΔCt) matrices for each gene (eq. (9)). Colored boxes indicate means, whiskers - 95% confidence intervals, letters within mean boxes - multiple t-test comparisons with significant differences at FDR < 0.05.

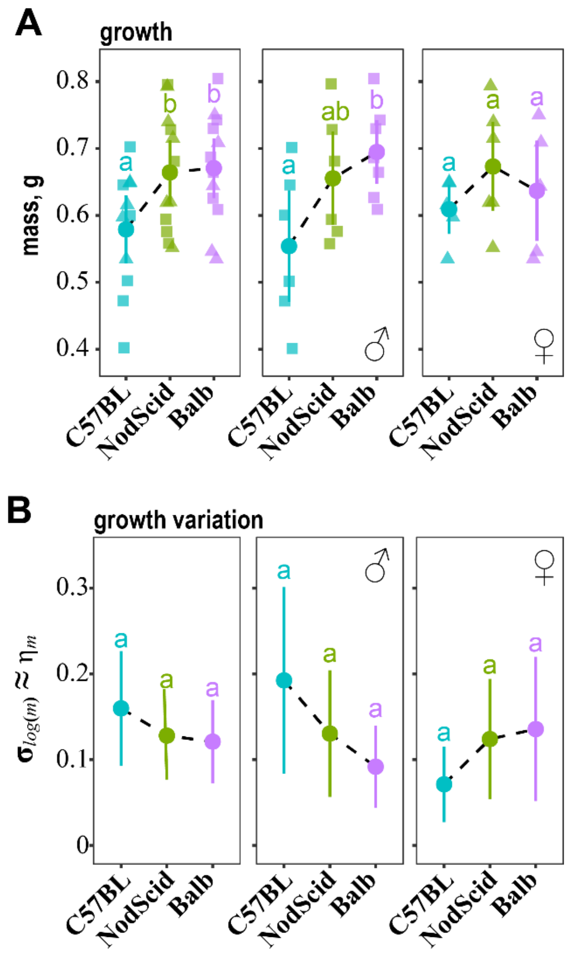


**Figure S3. The effect of surrogate mother MHC haplotype on C57BL/6J embryos mass and its fluctuation.**

**A)** Non-adjusted mass of C57BL/6J embryos developed in C57BL/6J, NOD-SCID, and BALB/c surrogate mothers; see Figure 3C.

**B)** GAMLSS estimations of $\hat{\sigma}_{log(m)} \approx \hat{\eta}_m$ for non-adjusted embryo mass; see Figure 3D.

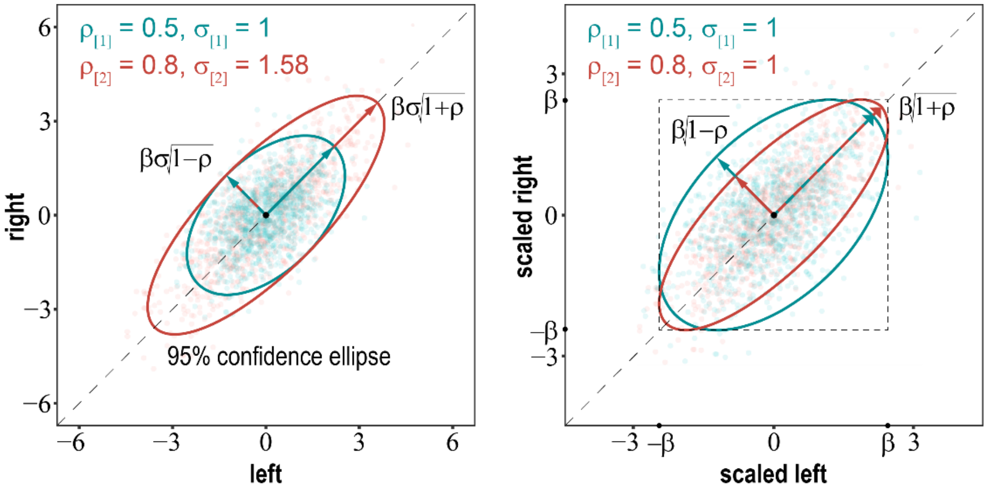


**Figure S4. Correlation is not a proper measure of the FA/"intrinsic" variability.**

To show that the correlation could be a misleading measure of the FA, let's assume a trait $x$ under two conditions [1] (green) and [2] (red). Under [1], the correlation between left and right $\rho_{l,r[1]} = 0.5$, and $\sigma_{x_{[1]}} = 1.0$; under [2], $\rho_{l,r[2]} = 0.8$ and $\sigma_{x_{[2]}} = \frac{\sigma_{x[1]}\sqrt{1-\rho_{l,r[1]}}}{\sqrt{1-\rho_{l,r[2]}}} = 1.58$.

Left panel, from the eq. (2), the "extrinsic" component of the DI of $x_{[1]}$ is greater than that of $x_{[2]}$:

$$\mathrm{Var}[\mathrm{E}(x_{[1]}|\ \xi)] \approx \frac{1}{2}\sigma^2_{x_{[1]}}\left(1 + \ \rho_{l,r[1]}\right) = 0.75, \text{ and } \mathrm{Var}[\mathrm{E}(x_{[2]}|\ \xi)] \approx 2.25.$$

From the eq. (3), the "intrinsic" component of the DI (FA) is equal for $x_{[1]}$ and $x_{[2]}$:

$$\mathrm{E}\left[\mathrm{Var}\left(x_{[1]}|\xi_{[1]}\right)\right] \approx \frac{1}{4}FA_{x_{[1]}} \approx \frac{1}{2}\sigma^2_{x_{[1]}}\left(1 - \rho_{l,r[1]}\right) = 0.25, \text{ and } \mathrm{E}\left[\mathrm{Var}\left(x_{[2]}|\xi_{[2]}\right)\right] \approx 0.25.$$

Ellipses correspond to 95% confidence ellipses, $\beta = \sqrt{\chi^2_{2,\alpha=0.05}} \approx 2.45$.

Right panel, estimation of the FA using only the correlation leads to a conclusion that $FA_{x_{[1]}} > FA_{x_{[2]}}$. The such discrepancy stems from that that correlation is estimated as the covariance of standardized variables. In other words, information on the fluctuation of $x$ ($\sigma_x^2$) is dropped off. Rewriting the FA for the standardized trait $x$: $FA_x \approx 2\left(1 - \rho_{l,r}\right)$ (eq. (2)), it is easy to see that omitting the changes in $\sigma_x^2$ could result in a misleading conclusion about changes in the $FA_x$.

**Table S1. Primers used for RT-qPCR**

| Gene | Description | Ensembl | Forward | Reverse |
|---|---|---|---|---|
| ***Actb*** | Actin, beta | ENSMUSG00000029580 | GGCTGTATTCCCCTCCATCG | CCAGTTGGTAACAATGCCATGT |
| ***Anxa5*** | Annexin A5 | ENSMUSG00000027712 | ATCCTGAACCTGTTGACATCCC | AGTCGTGAGGGCTTCATCATA |
| ***B2m*** | Beta-2 microglobulin | ENSMUSG00000060802 | TTCTGGTGCTTGTCTCACTGA | CAGTATGTTCGGCTTCCCATTC |
| ***Car2*** | Carbonic anhydrase 2 | ENSMUSG00000027562 | CGGATGGATTGGCTGTTTTGG | GCACGCTTCCCCTTTGTTTTA |
| ***Cd36*** | CD36 antigen | ENSMUSG00000002944 | ATGGGCTGTGATCGGAACTG | GTCTTCCCAATAAGCATGTCTCC |
| ***Col3a1*** | Collagen, type III, alpha 1 | ENSMUSG00000026043 | CTGTAACATGGAAACTGGGGAAA | CCATAGCTGAACTGAAAACCACC |
| ***Dcn*** | Decorin | ENSMUSG00000019929 | TCTTGGGCTGGACCATTTGAA | CATCGGTAGGGGCACATAGA |
| ***Gapdh*** | Glyceraldehyde-3-phosphate dehydrogenase | ENSMUSG00000057666 | AGGTCGGTGTGAACGGATTTG | TGTAGACCATGTAGTTGAGGTCA |
| ***G0s2*** | G0/G1 switch gene 2 | ENSMUSG00000009633 | AAGGAGATGATGGCGCAGAAG | GCTGCACACCGTCTCAACTA |
| ***Hdac2*** | Histone deacetylase 2 | ENSMUSG00000019777 | TGTGCTTGCCATCCTCGAATT | AGCTTCCTCAACACCATCACC |
| ***Hsp90ab1*** | Heat shock protein 90 alpha, class B member 1 | ENSMUSG00000023944 | TCAAACAAGGAGATTTTCCTCCG | GCTGTCCAACTTAGAAGGGTC |
| ***Postn*** | Periostin, osteoblast specific factor | ENSMUSG00000027750 | GAGGTCTCCAAGGTCACAAAGT | TGTGTCTCCCTGAAGCAGTCT |
| ***Rac1*** | RAS-related C3 botulinum substrate 1 | ENSMUSG00000001847 | GAGACGGAGCTGTTGGTAAAA | ATAGGCCCAGATTCACTGGTT |
| ***Rn18s*** | 18S ribosomal RNA | ENSMUSG00000119584 | CTCAACACGGGAAACCTCAC | CGCTCCACCAACTAAGAACG |

**Table S2. The number of implanted and live C57BL/6J embryos in experimental surrogate mother groups (mean ± SE)**

| Surrogate mother group | n | implanted embryo | live embryos |
|---|---|---|---|
| C57BL/6J | 13 | 5.92 ± 1.05 | 4.69 ± 1.01 |
| NOD-SCID | 16 | 4.25 ± 0.98 | 2.88 ± 0.75 |
| BALB/c | 13 | 4.69 ± 1.31 | 3,77 ± 1.06 |

**Table S3. GAMLSS models of the relationships between the distribution parameters (mean - $\mu$, dispersion - $\sigma$) of logarithm-transformed embryo mass ($log(m)$) and individual estimates of pulled "intrinsic" gene noise ($\eta_{int.}$) for C57BL/6J embryos**

**A) Models for $log(m)$**

| group | GAMLSS model | estimate ($\hat{\alpha}, \hat{\beta}$) ± *s.e.* | $p$ |
|---|---|---|---|
| all[(a)] | (1) $\mu_{log(m)} \sim \alpha_0 + \alpha log(\eta_{int.})$ | **-0.264±0.028** | **< 0.001** |
| | (2) $\sigma_{log(m)} \sim \beta_0 + \beta log(\eta_{int.})^{(b)}$ | **0.777±0.276** | **0.008** |

| | | | |
|---|---|---|---|
| C57BL/6J | (1) $\mu_{log(m)} \sim \alpha_0 + \alpha log(\eta_{int.})$ | **-0.208±0.024** | **< 0.001** |
| | (2) $\sigma_{log(m)} \sim \beta_0 + \beta log(\eta_{int.})$ | **3.524±1.168** | **0.019** |

| | | | |
|---|---|---|---|
| NOD-SCID | (1) $\mu_{log(m)} \sim \alpha_0 + \alpha log(\eta_{int.})$ | **-0.255±0.054** | **0.002** |
| | (2) $\sigma_{log(m)} \sim \beta_0 + \beta log(\eta_{int.})$ | 0.715±0.594 | 0.263 |

| | | | |
|---|---|---|---|
| BALB/c | (1) $\mu_{log(m)} \sim \alpha_0 + \alpha log(\eta_{int.})$ | **-0.241±0.046** | **0.001** |
| | (2) $\sigma_{log(m)} \sim \beta_0 + \beta log(\eta_{int.})^{)}$ | 0.877±0.534 | 0.139 |

**B) Models for litter-size adjusted $log(m)$**

| group | GAMLSS model | estimate ($\hat{\alpha}, \hat{\beta}$) ± *s.e.* | $p$ |
|---|---|---|---|
| all | (1) $\mu_{log(m)} \sim \alpha_0 + \alpha log(\eta_{int.})$ | **-0.196±0.021** | **< 0.001** |
| | (2) $\sigma_{log(m)} \sim \beta_0 + \beta log(\eta_{int.})^{)}$ | **0.979±0.247** | **< 0.001** |

| | | | |
|---|---|---|---|
| C57BL/6J | (1) $\mu_{log(m)} \sim \alpha_0 + \alpha log(\eta_{int.})$ | **-0.144±0.017** | **< 0.001** |
| | (2) $\sigma_{log(m)} \sim \beta_0 + \beta log(\eta_{int.})^{)}$ | **3.031±0.667** | **0.003** |

| | | | |
|---|---|---|---|
| NOD-SCID | (1) $\mu_{log(m)} \sim \alpha_0 + \alpha log(\eta_{int.})$ | **-0.24±0.039** | **< 0.001** |
| | (2) $\sigma_{log(m)} \sim \beta_0 + \beta log(\eta_{int.})^{)}$ | **1.309±0.449** | **0.015** |

| | | | |
|---|---|---|---|
| BALB/c | (1) $\mu_{log(m)} \sim \alpha_0 + \alpha log(\eta_{int.})$ | **-0.103±0.021** | **0.001** |
| | (2) $\sigma_{log(m)} \sim \beta_0 + \beta log(\eta_{int.})^{)}$ | **1.801±0.506** | **0.007** |

[(a)] - all embryos.

[(b)] – Note that from the Taylor expansion for the moments $\sigma_{log(m)} \approx \frac{\mu_m}{\sigma_m} = \eta_m$. Assuming a normal distribution of $log(m)$: $\sigma_{log(m)} = \sqrt{log(1 + \eta_m^2)}$, and for $\eta_m^2 < 1$, $\sigma_{log(m)} \approx \eta_m$.

**Table S4. GAMLSS models of the relationships between the distribution parameters ($\mu$, $\sigma$) of $log(PLGF)$ (PLGF concentrations, ng/l) and individual estimates of pulled "intrinsic" gene noise ($\eta_{int.}$) for C57BL/6J embryos**

| group | GAMLSS model | estimate ($\hat{\alpha}, \hat{\beta}$) ± *s.e.* | $p$ |
|---|---|---|---|
| all | (1) $\mu_{log(PLGF)} \sim \alpha_0 + \alpha log(\eta_{int.})$ | **-0.551±0.081** | **< 0.001** |
| | (2) $\sigma_{log(PLGF)} \sim \beta_0 + \beta log(\eta_{int.})$ | **1.424±0.314** | **< 0.001** |
| C57BL/6J | (1) $\mu_{log(PLGF)} \sim \alpha_0 + \alpha log(\eta_{int.})$ | **-0.555±0.092** | **0.001** |
| | (2) $\sigma_{log(PLGF)} \sim \beta_0 + \beta log(\eta_{int.})$ | 1.823±0.896 | 0.081 |
| NOD-SCID | (1) $\mu_{log(PLGF)} \sim \alpha_0 + \alpha log(\eta_{int.})$ | **-0.524±0.105** | **0.001** |
| | (2) $\sigma_{log(PLGF)} \sim \beta_0 + \beta log(\eta_{int.})$ | **2.338±0.555** | **0.003** |
| BALB/c | (1) $\mu_{log(PLGF)} \sim \alpha_0 + \alpha log(\eta_{int.})$ | **-0.353±0.089** | **0.004** |
| | (2) $\sigma_{log(PLGF)} \sim \beta_0 + \beta log(\eta_{int.})$ | 0.372±0.53 | 0.502 |
